# High potential for weathering and climate effects of non-vascular vegetation in the Late Ordovician

**DOI:** 10.1038/ncomms12113

**Published:** 2016-07-07

**Authors:** P. Porada, T. M. Lenton, A. Pohl, B. Weber, L. Mander, Y. Donnadieu, C. Beer, U. Pöschl, A. Kleidon

**Affiliations:** 1 Department of Environmental Science and Analytical Chemistry (ACES), Stockholm University, 10691 Stockholm, Sweden; 2 Bolin Centre for Climate Research, Stockholm University, 10691 Stockholm, Sweden; 3 Earth System Science Group, College of Life and Environmental Sciences, University of Exeter, Laver Building (Level 7), North Park Road, Exeter EX4 4QE, UK; 4 Laboratoire des Sciences du Climat et de l'Environnement, LSCE/IPSL, CEA-CNRS-UVSQ, Université Paris-Saclay, F-91191 Gif-sur-Yvette, France; 5 Max Planck Institute for Chemistry, PO Box 3060, 55020 Mainz, Germany; 6 Department of Environment, Earth and Ecosystems, The Open University, Milton Keynes, Buckinghamshire MK7 6AA, UK; 7 Aix-Marseille Université, CNRS, IRD, CEREGE UM34, 13545 Aix en Provence, France; 8 Max Planck Institute for Biogeochemistry, PO Box 10 01 64, 07701 Jena, Germany

## Abstract

It has been hypothesized that predecessors of today's bryophytes significantly increased global chemical weathering in the Late Ordovician, thus reducing atmospheric CO_2_ concentration and contributing to climate cooling and an interval of glaciations. Studies that try to quantify the enhancement of weathering by non-vascular vegetation, however, are usually limited to small areas and low numbers of species, which hampers extrapolating to the global scale and to past climatic conditions. Here we present a spatially explicit modelling approach to simulate global weathering by non-vascular vegetation in the Late Ordovician. We estimate a potential global weathering flux of 2.8 (km^3^ rock) yr^−1^, defined here as volume of primary minerals affected by chemical transformation. This is around three times larger than today's global chemical weathering flux. Moreover, we find that simulated weathering is highly sensitive to atmospheric CO_2_ concentration. This implies a strong negative feedback between weathering by non-vascular vegetation and Ordovician climate.

## Abstract (web summary)

Early non-vascular vegetation may have caused an interval of glaciations in the Late Ordovician by enhancing global chemical weathering. Here, by simulating the organisms with a spatially explicit, process-based model, the authors propose that Ordovician vegetation had a high potential for chemical weathering.

## Graphical abstract

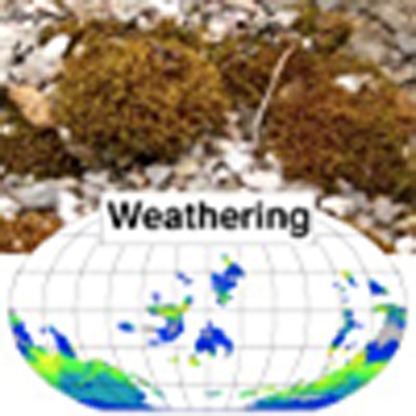

Lichens and bryophytes have been shown to significantly enhance weathering of the surface rocks on which they grow compared with abiotic conditions^1^,^2^,^3^. They increase both physical as well as chemical weathering rates: lichen rhizines and bryophyte rhizoids break up the rock surface, followed by dissolution of the minerals through different organic acids, alkalinolysis and chelating agents released by the organisms^4^.

Measurements of weathering intensity below lichens indicate an enhancement by around one order of magnitude compared with abiotic conditions for cool mountainous regions, such as Norway^5^, as well as for warm arid regions, for example, Lanzarote^6^. In moist tropical regions an enhancement of two orders of magnitude has been observed^7^,^8^. These measurements are complemented by studies based on mini-watersheds in the northeastern United States^9^,^10^, which quantify directly the amount of chemical elements exported from the rock and which confirm the enhancement effect (factor 2–16). Bryophytes, too, have been proven to enhance weathering significantly under field conditions in Canada^1^ as well as in microcosms^2^,^11^.

At the global scale, however, it is difficult to assess the role that lichens and bryophytes play in weathering based on these local findings. The global distribution of rocks covered by lichens and bryophytes is poorly known^12^. Several studies have concluded that lichens and bryophytes could not significantly enhance the global weathering flux, since vascular plants show a considerably higher enhancement of weathering^2^,^13^. Other studies, however, suggest that enhancement of weathering by lichens and bryophytes is still sufficiently large for global effects^3^,^11^.

The uncertainty regarding the relative importance of lichens and bryophytes for the global weathering flux limits climate reconstructions of the geological past^3^. On the basis of field observations, it has been concluded that the emergence of large vascular plants with rooting systems in the Devonian led to a large increase in global chemical weathering rates^14^. Including this effect in a global geochemical model^14^ causes a decrease in atmospheric CO_2_ based on the well-known silicate-weathering feedback^15^.

Predecessors of today's bryophytes, however, could also have enhanced chemical weathering. Molecular phylogenies support the traditional view that non-vascular (bryophyte) plant lineages (liverworts, hornworts and mosses) all pre-date the origin of vascular plants^16^. The ancestors of modern brypophytes are thought to have evolved during the Mid-Late Ordovician period based on the microfossil record of spores^16^ and improvements in molecular clock dating support an early origin^16^. The oldest lichen fossil found so far, exhibiting symbionts and anatomy similar to extant lichens, dates to the Early Devonian^17^; however, lichens could be much older, as shown by a potential symbiosis of fungal hyphae and cyanobacteria from the Late Precambrian^18^. Thus, early non-vascular plants, together with lichens, could also have led to a drawdown in atmospheric CO_2_.

It has been hypothesized that the interval of glaciations at the end of the Late Ordovician (peaking in the Hirnantian Stage) could be explained by a decrease in atmospheric CO_2_ because of the biotic enhancement of weathering^11^. Therefore, microcosms containing moss growing on mineral surfaces were used to estimate the enhancement of weathering compared with microcosms without moss. The results indicate a considerable increase in weathering for several elements, with highest values for iron (over two orders of magnitude) and phosphorus, with calcium and magnesium weathering amplified by factors of circa 1.5–5 across different silicate rock types.

In order to translate the increase in chemical weathering rates into a decrease in atmospheric CO_2_, the results from the microcosm experiment were used to calibrate an enhancement factor in a global biogeochemical model^11^. Such an approach neglects the spatiotemporal variation of the global moss cover that results from climate patterns and that clearly controls weathering by moss. Moreover, the enhancement factor is based only on one species of moss, growing in the modern atmosphere, while weathering by lichens and bryophytes has been shown to be species-specific^1^,^19^.

In this study, we present a complementary approach, which derives chemical weathering by lichens and bryophytes in the Ordovician from their net primary productivity (NPP), which is calculated by a novel, global process-based non-vascular vegetation model that simulates multiple, physiologically different, artificial species^20^. To translate NPP into a weathering flux it is assumed that lichens and bryophytes dissolve surface rocks to access phosphorus for the build-up of new biomass^21^. The amount of rock that is weathered can then be estimated from the phosphorus equivalent of NPP^22^ and the phosphorus concentration of rocks. To account for the limited supply of unweathered rock material in shallow regions, we cap biotic weathering at the erosion rate. Furthermore, we constrain weathering by the transport capacity of runoff for dissolved weathered material. Since these limitations on weathering result in a lower nutrient supply from rocks, we reduce our estimate of NPP accordingly. By representing the species-specific, spatiotemporal dynamics of weathering by lichens and bryophytes in the Late Ordovician, we assess the significance of these organisms for Ordovician weathering and climate. Throughout the manuscript, we use the term chemical weathering to describe the partial dissolution and chemical transformation of a certain volume of primary minerals into solutes and secondary minerals. This includes all elements, such as calcium, phosphorus, iron and so on, in the primary minerals. We use volume as a basis to compare the rate of chemical weathering to the erosion rate.

## Results

### Weathering by lichens and bryophytes in the Ordovician

**Figure 1:**
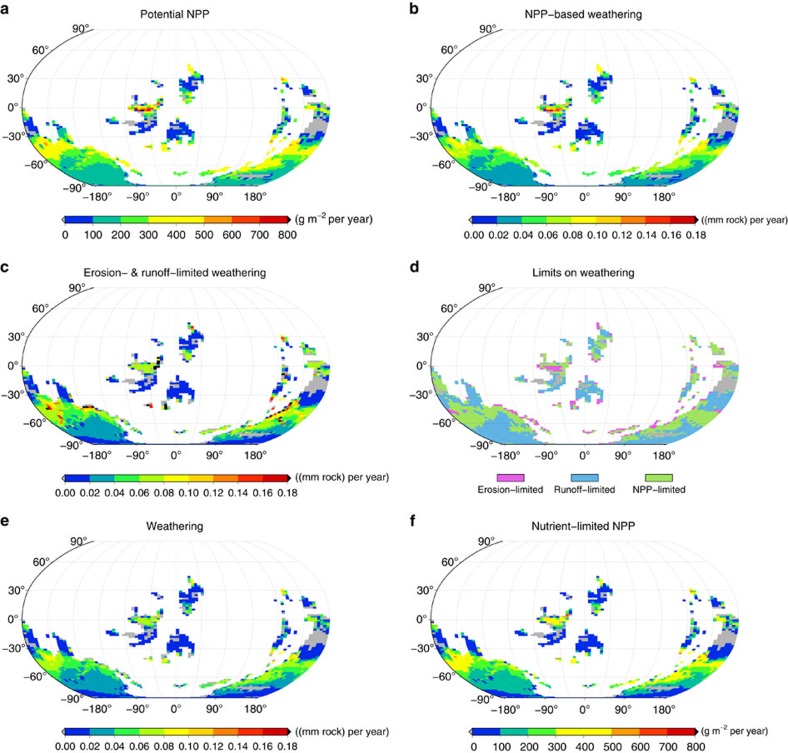
Global patterns of NPP and weathering. (**a**) Potential NPP simulated by the lichen and bryophyte model for the Late Ordovician, 450 million years ago (450 Ma). (**b**) NPP-based weathering, (**c**) weathering based on the minimum of erosion and transport of solutes by runoff, (**d**) limiting factors on weathering, (**e**) weathering constrained by NPP, erosion and runoff and (**f**) NPP limited by nutrient supply from weathering. The maps show average values over the last 50 years of a 600-year simulation with 300 initial species. The atmospheric CO_2_ concentration is 2,240 p.p.m. or 8 PAL. The grey areas denote regions where no species are able to survive. For the small grey area near the south pole the lichen and bryophyte model simulates a small ice sheet.

The global pattern of chemical weathering by Ordovician lichens and bryophytes, their NPP and their surface coverage as well as the pattern of the limiting factors on weathering are shown in Fig. 1. The estimates are obtained from a baseline simulation of the non-vascular vegetation model, run at eight preindustrial atmospheric level(PAL; 1 PAL=280 p.p.m.) of atmospheric CO_2_ and 14% of atmospheric O_2_ for 600 years to reach steady state, with an initial number of 300 species (see Methods section for details).

Potential NPP of lichens and bryophytes depends only on climatic factors (Fig. 1a). It shows a latitudinal pattern, with maximum values at the equator and high values in the region between 30° and 60°. Between the equator and 30° N and 30° S, respectively, NPP is low and in some regions none of the simulated lichen and bryophyte species are able to survive. This distribution can be explained by water limitation of NPP in (sub)tropical regions and limitation by low temperature and light availability in polar regions (see Methods section). The global pattern of weathering by lichens and bryophytes based solely on their NPP is identical to that of potential NPP, since the conversion factors used to translate NPP into a weathering flux are assumed to be globally uniform (Fig. 1b).

Chemical weathering constrained by both the erosion rate and the transport capacity of runoff for dissolved weathered material is strongest in regions of pronounced relief and high rainfall (Fig. 1c). In areas of flat topography, weathering is limited by erosion, which is directly related to the mean surface elevation above the sea level. In arid regions, weathering is limited by low runoff. The global pattern of weathering thus results from the minimum of the limiting factors NPP, erosion and runoff (Fig. 1d,e). In areas where NPP-based weathering exceeds erosion- and runoff-limited weathering, the supply of phosphorus from rocks is not sufficient to sustain potential NPP, which is consequently reduced (Fig. 1f). The strongest reduction of potential NPP occurs at the equator, because of low erosion rates, and in dry regions at 30° S and at high latitudes, because of low rainfall. At the global scale, NPP-based weathering of 3.6 (km^3^ rock) yr^−1^ is reduced by 22% to a realized weathering of 2.8 (km^3^ rock) yr^−1^. This is accompanied by a reduction in NPP because of phosphorus limitation in shallow or arid regions. Potential NPP of 18.7 (Gt C) yr^−1^ is reduced by 23% to 14.4 (Gt C) yr^−1^ of realized NPP.

Global annual values of lichen and bryophyte weathering, NPP, gross primary productivity (GPP), surface coverage and biomass are listed in Table 1 together with the corresponding values for today's climate in the presence of vascular plants. Today's values were calculated by forcing the lichen and bryophyte model with data fields for today's climate and other current environmental conditions^20^. In addition, shown in Table 1 are observation-based estimates of NPP, GPP, surface coverage and biomass of today's total terrestrial vegetation and total, that is, biotic plus abiotic, global chemical weathering.

Considering only lichens and bryophytes, all values corresponding to the Ordovician are considerably larger than those for today's climate. The relative increase differs between the variables. NPP and GPP increase by a factor of around six, cover and biomass by around three and weathering increases much more by a factor of ∼60.

Chemical weathering by Ordovician lichens and bryophytes is more than three times larger than today's combined abiotic and biotic global chemical weathering. Values of NPP and GPP of Ordovician lichens and bryophytes, however, only amount to ∼25% of today's total terrestrial vegetation, while Ordovician biomass and vegetation surface coverage are 30% and 60% of today's values, respectively. These results are explained below in the Discussion section.

In Table 1 we show today's combined abiotic and biotic global chemical weathering rate. It is estimated that vegetation enhances chemical weathering by a factor of ∼4–7 (refs 14, 23). Therefore, the combined rate should be a good approximation for weathering by vegetation alone, which includes vascular plants, bryophytes and lichens.

### Sensitivity of weathering to environmental factors

**Figure 2:**
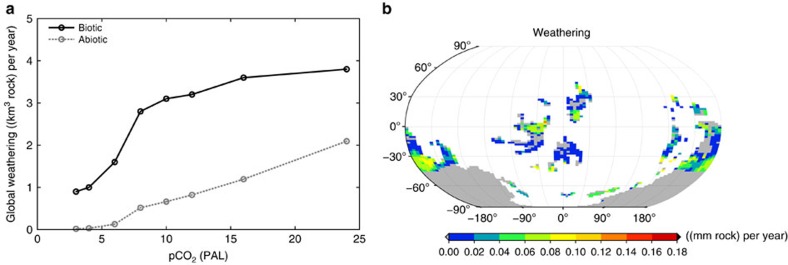
Sensitivity of weathering to environmental factors. (**a**) Global chemical weathering as a function of atmospheric CO_2_ concentration. One PAL corresponds to 280 p.p.m. Each dot represents a model simulation at the respective atmospheric CO_2_ concentration. The black solid line shows weathering enhanced by non-vascular vegetation derived from a 600-year simulation with 300 initial species. The grey dashed line represents abiotic weathering, calculated by a GEOCARB approach. (**b**) Effect of a continental-sized ice sheet south of 30° S on the global pattern of weathering for the Late Ordovician (450 Ma), derived from NPP of lichens and bryophytes constrained by erosion and runoff. The map shows average values over the last 50 years of a 600-year simulation with 300 initial species. The atmospheric CO_2_ concentration is 2,240 p.p.m. (8 PAL). The grey areas denote regions where no species are able to survive, notably including the ice sheet mask prescribed from the South Pole to the mid-latitudes.

Estimated global chemical weathering by lichens and bryophytes in the Ordovician shows a strong dependence on atmospheric CO_2_ concentration, as illustrated in (Fig. 2a). The increase in weathering with increasing CO_2_ results from the positive effect of high atmospheric CO_2_ concentrations on NPP. While the relation looks exponential at values of CO_2_ lower than 8 PAL, it seems to change at high values to a logarithmic one. Below we discuss possible reasons for the strong dependence of NPP on atmospheric CO_2_.

We, furthermore, show weathering for abiotic conditions in (Fig. 2a), which is calculated using the GEOCARB III model^14^. Abiotic weathering is in general considerably lower than weathering enhanced by non-vascular vegetation. However, abiotic weathering does not show saturation at very high values of CO_2_, in contrast to biotic weathering.

To account for the existence of a continental-sized ice sheet south of 30° S in the Hirnantian Stage^24^, an additional vegetation model run is performed with a corresponding glacier mask and updated climatic forcing fields (Fig. 2b). Although the ice sheet does not cover the regions of highest NPP and weathering at the equator and close to 30° S, it covers vast regions of Gondwana and, therefore, reduces weathering considerably from 2.8 to 1.5 km^3^ yr^−1^ and NPP from 14.4 to 7.6 Gt yr^−1^.

### Sensitivity of weathering to number of artificial species

**Figure 3:**
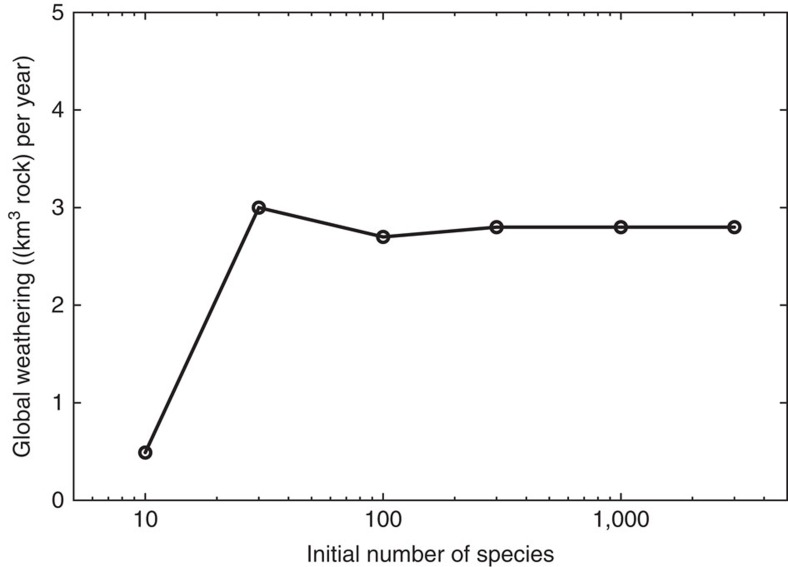
Sensitivity of weathering to number of species. Global chemical weathering by lichens and bryophytes as a function of the initial number of species in a simulation conducted for the Late Ordovician at 8 PAL. Each dot represents a model simulation carried out at the corresponding initial species number.

Figure 3 shows that global weathering by lichens and bryophytes increases with the number of initial artificial species used in a simulation and remains constant for larger numbers of initial species. This results from an undersampling of the very large space of possible combinations of physiological parameters. Species that are capable of high NPP and, consequently, strong weathering, are probably not generated if the number of initial species is too low. This means that, at the beginning of the colonization of the land surface by non-vascular plants, biotic weathering could have been lower because of very low diversity and productivity.

## Discussion

In this study we estimate chemical weathering in the Late Ordovician by early forms of lichens and bryophytes. We quantify their NPP during this period with a process-based vegetation model and then translate the NPP into a weathering flux^22^, additionally accounting for limitation of weathering by low erosion and runoff.

Our main finding is a huge weathering potential of Ordovician lichens and bryophytes, more than three times higher than today's combined abiotic and biotic global chemical weathering flux. This value results from a 60-fold increase in weathering by these organisms compared with today's value (Table 1), which could be explained by the following factors: first, lack of vascular plants in the Ordovician allows lichens and bryophytes to cover the entire area available for growth of vegetation. Second, Ordovician lichens and bryophytes have increased access to mineral surfaces because of the absence of competing weathering by vascular plants. Finally, high Ordovician atmospheric CO_2_ has a strong increasing effect on NPP of lichens and bryophytes and, consequently, on NPP-based weathering.

**Figure 4:**
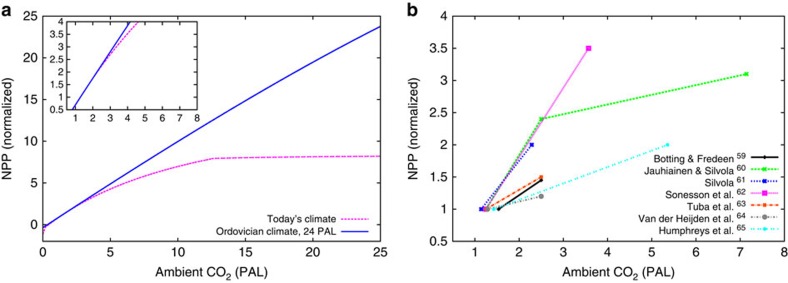
Effect of elevated CO_2_ on NPP. (**a**) NPP calculated by the model for constant light, temperature and water content. The blue solid line corresponds to a species adapted to Ordovician climate and an atmospheric CO_2_ concentration of 24 PAL and the dashed magenta line corresponds to a species adapted to today's climate and CO_2_ concentration. (**b**) NPP of lichens and bryophytes measured in field or laboratory experiments as a function of ambient CO_2_. Each line stands for a different study and each dot represents a measurement of NPP at the corresponding CO_2_ concentration. NPP is normalized in order to make the CO_2_ response comparable between different species. The inset figure in **a** focuses on the range of ambient CO_2_ shown in **b**.

In the following, we discuss the plausibility of our estimates, starting with the large value of NPP for the Ordovician, which is ∼25% of today's total terrestrial NPP^25^. The main factor for such a high productivity is the strong CO_2_ sensitivity of simulated NPP, combined with high levels of atmospheric CO_2_ in the Ordovician. The CO_2_ response can be analysed in the model for individual artificial species, by varying ambient CO_2_, but otherwise keeping constant boundary conditions, such as water content, temperature and radiation. To find appropriate species, we choose two regions with similar climate, one from a simulation with today's climatic and CO_2_ forcing and another from an Ordovician simulation with a high atmospheric CO_2_ value of 24 PAL. From these two regions we select the dominant species, shown in (Fig. 4a). It can be seen that for the high Ordovician CO_2_ level, the model favours a species that has a higher photosynthetic capacity than the species dominant under today's CO_2_, given otherwise similar climatic conditions. This seems plausible from the viewpoint of natural selection and leads to a higher NPP in the Ordovician simulation. Most dynamic vegetation models, however, do not implement such a large physiological flexibility, but rely on a few plant functional types with fixed photosynthesis parameters. They cannot adapt to strongly differing environmental conditions. Hence, we attribute the strong sensitivity of NPP to CO_2_ to the physiological flexibility of our model.

Experiments confirm that the CO_2_ sensitivity of lichens and bryophytes is species-specific (Fig. 4b). The species differ with regard to the slope of the CO_2_ response as well as the ambient CO_2_ concentration, at which photosynthesis saturates. We want to point out that Fig. 4 does not represent an evaluation of the model, it rather illustrates the large variety in CO_2_ responses of different lichen and bryophyte species. This supports the possibility of high NPP of early lichens and bryophytes in the Late Ordovician.

Two of the experimental studies considered here measure NPP of bryophytes at CO_2_ concentrations up to 8 PAL (see also Methods section). They find a two to threefold increase in NPP, whereas our artificial species from today shows a sixfold increase, relative to NPP at 360 p.p.m. of CO_2_. This may be explained by the fact that the artificial species has a lower photosynthetic capacity than the bryophytes from the experiments, maybe to avoid high respiration costs. As a consequence, NPP of the artificial species increases only slowly with rising ambient CO_2_ leading to a much lower NPP at 360 p.p.m. of CO_2_, compared with the bryophytes (see Methods section for details). For the same reason, however, NPP of the artificial species saturates at a higher CO_2_ concentration than the bryophyte, leading to a larger CO_2_ sensitivity. We do not want to exclude the possibility that our model is too flexible regarding the shape of the CO_2_ response. We therefore test a minimum CO_2_-sensitivity scenario, where potential NPP is reduced by 50%. Since weathering depends also on environmental limits in our approach, this translates into a 40% reduced estimate of global chemical weathering for the Ordovician of 1.6 (km^3^ rock) yr^−1^.

Several studies substantiate the occurrence of high non-vascular NPP in the geological past. On the basis of estimated Ordovician soil CO_2_ production, it has been concluded that the above-ground productivity fuelling this CO_2_ production must have been similar to today's (ref. 26). Hence, non-vascular productivity could have been as high as the vascular one. The course of *δ*^13^C through the Phanerozoic shows a marked increase in the Late Ordovician consistent with a new source of organic carbon on land and concomitant increase in global organic carbon burial^27^. Moreover, pre-vascular organic carbon deposits were already large, although they are not in the form of coal^3^. The occurence of coal before the Devonian is unlikely, albeit difficult to assess because of the sparse occurence of terrestrial sediments preserved from before the Devonian^16^.

An alternative estimate of NPP for the Ordovician^28^ has been made by multiplying today's global cryptogamic productivity on ground^12^ with a value of 1.8, which is the ratio of today's total vegetated area to the area covered by cryptogams. The estimated value of 4.3 (Gt C) yr^−1^ is markedly lower than our estimate, which can be explained by simplifying assumptions in this study, such as a linear relationship between area and productivity and no CO_2_ fertilization.

**Figure 5:**
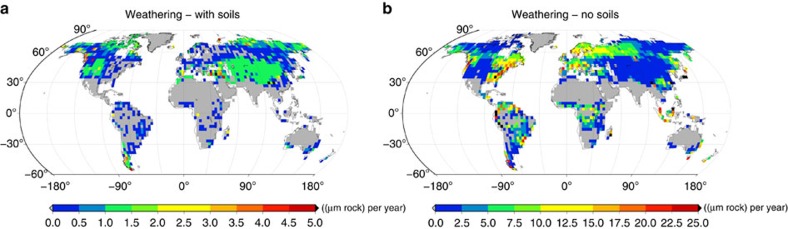
Weathering for today's climate. Global pattern of chemical weathering by lichens and bryophytes for today's climate, constrained by their NPP, erosion and runoff. (**a**) NPP-based weathering is constrained in each grid cell to the fraction of bare rock surface. (**b**) NPP-based weathering may take place on the whole land surface area. The map shows average values over the last 50 years of a 600-year simulation with 300 initial species. Runoff is derived from annual average values of climate variables^20^. Note the differing ranges of the colour scales.

Our estimate of realized NPP for the Ordovician is around six times higher than today's NPP of lichens and bryophytes. However, simulated chemical weathering by the organisms is enhanced by a factor of 60 in the Late Ordovician compared with today's value. We suggest that the absence of vascular plants in the Ordovician explains the remaining part of the difference in chemical weathering rates. Vascular plants are likely more efficient in enhancing weathering rates than lichens and bryophytes^2^,^29^. As a consequence, lichens and bryophytes are usually separated from unweathered rock material by a layer of soil in ecosystems that are dominated by vascular plants, such as forests and grasslands. In these ecosystems, growth of lichens and bryophytes on bare rocks is confined to boulders and cliffs, which cover only a small fraction of the land surface. This strongly limits the contribution of lichens and bryophytes to total chemical weathering. In other ecosystems, such as cold and warm deserts, lichens and bryophytes often are the dominant vegetation. However, low runoff largely limits chemical weathering by the organisms in these regions. To assess the effect of soils on weathering by today's lichens and bryophytes, we assume that all simulated growth in the model may lead to weathering, not only the part that takes place on bare soil. This increases weathering by today's lichens and bryophytes by a factor of ∼10, which, in combination with increased atmospheric CO_2_, explains the strong increase in weathering from today to the Late Ordovician (Fig. 5).

Given the large value of today's total terrestrial NPP compared with the Ordovician, the current global chemical weathering flux appears to be quite small (Table 1). This implies that, for a given amount of biotic weathering, today's vascular vegetation exhibits a much higher productivity than Ordovician lichens and bryophytes at the global scale. We explain this finding by a high nutrient recycling ratio of modern ecosystems of ∼50:1 (ref. 30), which results from the deep rooting systems of vascular plants and associated mycorrhiza. Although lichens and bryophytes are able to resorb phosphorus from senescing tissue, they most likely cannot access phosphorus that has been leached or released from decaying biomass and transported by water to deeper soil layers. In our model, we therefore assume a low recycling ratio of 5:1 for Late Ordovician ecosystems. This value is based on measurements of both weathering rates of lichens on rocks as well as their productivity in desert regions^20^ (see Methods section). Our estimates of chemical weathering and NPP are sensitive to the recycling ratio. In the unlikely case that Ordovician ecosystems had the same high recycling ratio of 50:1 compared with modern ecosystems, weathering decreases to 0.34 (km^3^ rock) yr^−1^ and NPP increases to 17.6 (Gt C) yr^−1^. For a value of 1:1, meaning no recycling, weathering increases to 3.7 (km^3^ rock) yr^−1^ because of the organisms' higher phosphorus requirement, while NPP is reduced to 3.9 (Gt C) yr^−1^ owing to nutrient limitation.

It is likely that vascular plants are more efficient in enhancing weathering than lichens and bryophytes^2^,^29^. However, our estimate of global chemical weathering in the Late Ordovician is higher than today's weathering (Table 1). One explanation is that the environmental limits, erosion rate and runoff allowed for a much higher global chemical weathering rate in the Late Ordovician compared with today's value. Thus, weathering by vascular plants today may be largely limited by environmental factors, and not by the ability of the plants to enhance weathering. In parts of the Amazon basin, for instance, the weathering capacity of the vegetation is much higher than the weathering allowed by the low erosion rate, leading to thick, highly weathered soils, but a relatively low value of chemical weathering^31^,^32^. In contrast, chemical weathering in the Ordovician is limited by NPP in many regions (Fig. 1). This means that hypothetical vascular vegetation in the Ordovician could increase weathering to a value of 3.8 (km^3^ rock) yr^−1^, which is the maximum chemical weathering possible under erosion- and runoff limitation.

When early lichens and bryophytes colonized the land surface in the Ordovician, they could well have used their full potential to enhance chemical weathering, until weathering became limited by erosion in many regions and a certain amount of organic material including phosphorus had accumulated in some ecosystems, facilitating a certain degree of recycling. This rationale supports a scenario of strong weathering by lichens and bryophytes leading to a large, but temporally limited release of phosphorus into the oceans and thereby to a short period of cooling because of enhanced marine productivity^11^. The short duration of the phosphorus release is explained by an ecosystem shift to more efficient nutrient recycling caused by nutrient limitation.

Both the erosion- and the runoff-based limits to chemical weathering are maximum rates, which means that the true rate of chemical weathering may be lower for some reason, such as a strong kinetic control on the weathering reactions because of low residence time of water in the soil, for instance. Several observations indicate, however, that reaction kinetics are often not the limiting factor for chemical weathering at the global scale^33^. First, chemical weathering does not show a very clear dependence on temperature at large scale. Second, chemical weathering increases with runoff for low and intermediate values of runoff, which should not be the case if the weathering reaction is far from equilibrium. Finally, chemical weathering rates determined in laboratory experiments exceed those measured in the field by up to two orders of magnitude, which suggests that factors other than reaction kinetics may limit weathering rates in many regions of the world. However, in some areas, such as humid mountainous regions, kinetic limitation of weathering reactions may be important. This is also supported by our alternative estimate of today's global chemical weathering of 1.3 (km^3^ rock) yr^−1^ based on the GEM-CO2 model, which is smaller than the 1.8 (km^3^ rock) yr^−1^ from our limit-based approach. To account for this potential overestimation, we assume that the relations between runoff, lithology and chemical weathering established in GEM-CO2 for today are the same for the Ordovician. We then scale our estimate of Ordovician weathering by the ratio of today's estimates from GEM-CO2 and the limit-based approach. This would reduce global chemical weathering in the Ordovician by 28% to 2.0 (km^3^ rock) yr^−1^.

The weaker increase in weathering and NPP at high CO_2_ values in (Fig. 2a) is because of the saturating Michaelis–Menten kinetics of the Farquhar scheme. Abiotic weathering, in contrast, does not show saturation. It increases almost linearly with atmospheric CO_2_ because of an exponential temperature dependence, which is largely compensated by the logarithmic dependence of temperature on CO_2_. The stronger than linear increase in biotic weathering and NPP for low CO_2_ values is probably due to the retreat of continental ice sheets at rising CO_2_ and a large increase in global land surface temperature. In addition, abiotic weathering shows a nonlinear response to increases in atmospheric CO_2_ below values of 8 PAL, since the relation between CO_2_ and land surface temperature is not logarithmic anymore at low CO_2_ values. The reason for this is a nonlinear increase in sea-surface temperatures with CO_2_ because of the complex ocean dynamics in the Fast Ocean Atmosphere Model (FOAM)^34^, which forces the Laboratoire de Météorologie Dynamique Zoom (LMDZ) atmospheric circulation model and, thereby, land surface temperature. The continental ice sheets result from a positive snow balance in the lichen and bryophyte model, which is calculated as a function of snowfall, surface temperature and a rate of lateral glacier movement. This snow scheme, however, is calibrated for the Holocene. For 8 PAL of CO_2_, for instance, it estimates only a small ice sheet near the south pole (Fig. 1), while there are indications that the ice sheet was much larger^24^. Given the large uncertainty concerning the extent of the Ordovician ice sheet, we think that using the default snow scheme in the model is appropriate.

Our results indicate that weathering by lichens and bryophytes is sensitive to the diversity of plants growing on the land surface (Fig. 3), and this raises the possibility that fluctuations in the diversity of lichens and bryophytes through geological time could have major biogeochemical consequences. Owing to a lack of macroscopic plant remains in rocks of Ordovician age, plant diversity at this time can be examined using fossilized spores^35^. These fossils provide the first widely accepted evidence for land plants during the Dapingian-Darriwilian interval^36^, and preserve evidence of an adaptive radiation of vascular plants in the Palaeozoic^37^. In light of our modelling results, shown in Fig. 3, we suggest that the magnitude of the weathering flux could have increased through the Ordovician as a consequence of the evolutionary radiation of primitive land plants. Thereby, our study highlights a possible link between the process of diversification and the biogeochemical processes that regulate Earth's climate.

Fossil cryptospores are not sufficiently abundant in the fossil record at this time to permit a detailed comparison between simulated vegetation and data on ancient vegetation derived from fossils. Nevertheless, recent work has demonstrated that cryptospores are found on all major Ordovician palaeocontinents and that, consequently, early land plants showed a global distribution before the Silurian^38^. Our simulations produce terrestrial vegetation that has such a global distribution (Fig. 1a), and in this respect our simulations are compatible with the available palaeobotanical data^37^,^38^.

In summary, our estimates imply that the Ordovician non-vascular biosphere had a similarly high capacity for biotic weathering than today's biosphere. That should be taken into account for understanding and modelling palaeoclimatic evolution. The colonization of the land by early forms of lichens and brypohytes in the Mid-Late Ordovician^16^,^39^ could have caused a large decrease in atmospheric CO_2_ concentration at the end of the Ordovician through the enhancement of silicate weathering.

The results from this study could be used as a basis for further analyses of the feedback between weathering and climate in the geological past. By using a coupled biogeochemical model, the effect of weathering on atmospheric CO_2_ concentration could be quantified. Subsequently, the CO_2_ sensitivity of weathering by lichens and bryophytes derived here could be used to estimate the impact of changing CO_2_ and climate on the biotic enhancement of weathering.

## Methods

### The non-vascular vegetation model

The process-based non-vascular vegetation model used in this study is a stand-alone vegetation model that uses gridded climatic fields to compute NPP as the difference between photosynthesis and respiration^20^. Similar to many dynamical vegetation models, photosynthesis is calculated by the Farquhar scheme and respiration via a Q_10_ relationship. These approaches are combined with lichen/bryophyte-specific characteristics, for example, a passive control of the water status (poikilohydry) or the decrease in CO_2_ diffusivity with increasing water content. In contrast to most global vegetation models, which use plant functional types, the lichen and bryophyte model explicitly represents physiological variation: the ranges of observed values of physiological parameters are sampled by a Monte-Carlo algorithm to generate artificial species. These species do not correspond directly to certain lichen and bryophyte species in the real world, but we assume that they represent the diverse physiological strategies of real lichens and bryophytes. This is a novel approach to include physiological variation in models of terrestrial vegetation, which is also used, for example, in the successful Jena Diversity-Dynamic Global Vegetation Model^40^.

The model is run for all species at each grid point of the climatic fields and the performance of the different species is analysed. Those species that cannot maintain their biomass are assumed to die out at the respective grid point and the remaining ones are used to compute NPP. This is performed by weighting each surviving species by its NPP accumulated over the simulation.

Note that processes related to uptake and loss of nutrients are not explicitly modelled. Instead, it is assumed that the costs of weathering agents and nitrogen fixation are included in the respiration term, which is based on observed values.

### Ordovician climate and environmental factors

The lichen and bryophyte model predicts surface coverage and NPP as a function of the following climate variables: downwelling shortwave and longwave radiation, rainfall, snowfall, air temperature, wind speed and relative humidity. These variables are calculated by the atmospheric general circulation model LMDZ^41^ for the Late Ordovician, 450 million years ago.

**Figure 6:**
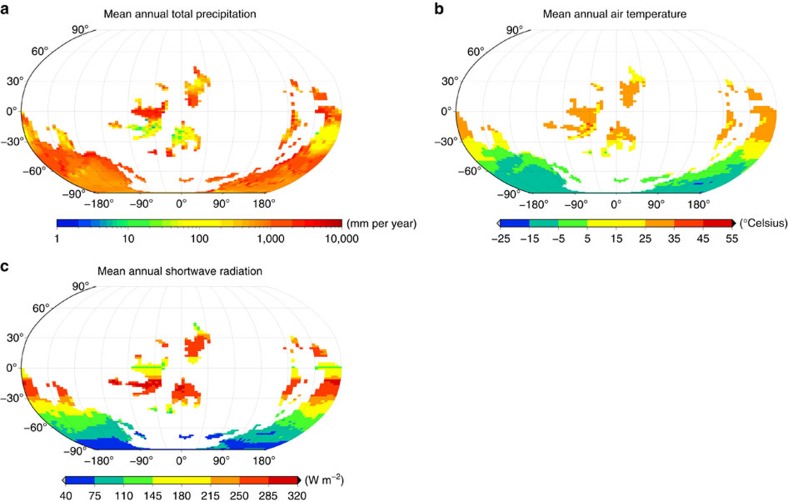
Simulated Ordovician climate. Global patterns of (**a**) total precipitation, (**b**) near-surface air temperature and (**c**) shortwave radiation for the Late Ordovician (450 Ma). The atmospheric CO_2_ concentration is 2,240 p.p.m. (8 PAL). The maps show average values over the whole time series of the interpolated climate variables that are derived from the monthly LMDZ output.

The LMDZ model is an Intergovernmental Panel on Climate Change-class atmospheric general circulation model benefitting, as the atmospheric component of the state-of-the-art Institut Pierre Simon Laplace (IPSL) Earth system model, from the latest physical and dynamical refinements. A mid-resolution version of the model is used in this study, providing a resolution of 3.75° × 1.875° with 39 vertical levels (21 in the troposphere) on a rectangular grid. LMDZ is forced with sea-surface temperatures from the Fast Ocean Atmosphere Model (FOAM^42^), also run at 450 Ma, to account for the effects of coupled ocean, atmosphere and sea ice. The palaeogeography for these simulations corresponds to the Blakey configuration^24^. Further details about the FOAM set-up can be found in a previous study^34^. LMDZ is run for 20 years to reach the atmospheric steady state. The climate variables needed by the lichen and bryophyte model are provided in the form of monthly values averaged over the last 5 years of the LMDZ simulation, see Fig. 6 for examples. A comparison of the areas of simulated low rainfall and high temperature with the spatial distribution of indicators of arid climate^43^ shows reasonable agreement.

The lichen and bryophyte model runs on an hourly time step to resolve fast processes such as dew formation, which are important for correctly simulating the organisms' NPP. Hence, to make the monthly climate variables from LMDZ usable for the lichen and bryophyte model, they are interpolated to hourly variables by a stochastic weather generator that was developed for this study (see Supplementary Fig. 1 and Supplementary Note 1).

In addition to the climatic fields, the lichen and bryophyte model requires information about the fraction of surface area that is available for growth, the frequency of disturbance events, such as fires, and the roughness length of the surface. The latter is needed to compute fluxes of heat and water between the lichen or bryophyte surface and the atmosphere. For the Ordovician, we assume that the whole land surface area is available for growth of lichens and bryophytes, except for regions covered by glaciers. We know little about the disturbance regime in the Ordovician, other than that there is no evidence of fires in the form of fossil charcoal, suggesting that oxygen was less than 17% of the atmosphere. Hence, we choose a relatively large disturbance interval of 100 years, which is a typical value for the boreal forest biome. The surface roughness length is set to a value of 0.01 m, corresponding to a desert or tundra ecosystem^20^.

### Model set-up

As baseline simulation for this study, the lichen and bryophyte model is run with the interpolated hourly climatic fields from LMDZ and an atmospheric CO_2_ content of 8 PAL, which equals 2,240 p.p.m. This CO_2_ level is close to the threshold required to trigger Late Ordovician glaciations in several climate models^44^. On the basis of the absence of fossil charcoal in the Ordovician and global geochemical modelling studies^45^, atmospheric O_2_ is set to 14% in our simulations. The initial number of artificial species is set to 300, since this amount of species sufficiently represents physiological variation (see Fig. 3). The model is run for 600 years to achieve a state where the number of surviving strategies remains constant for more than 100 years. We assume that this is a sufficient condition for a steady state. Since only 1 year of interpolated LMDZ output is available, we repeat this year 600 times. The estimates of the study are based on the averaged output of the last 50 years of the simulation.

### Derivation of chemical weathering from NPP

To derive chemical weathering by lichens and bryophytes from their NPP, we use a method from a previous study^22^. First, the phosphorus requirement associated with a given NPP is calculated from the C:P ratio of lichen and bryophyte biomass. This phosphorus requirement is then translated into a potential uptake of phosphorus from surface rocks, thereby accounting for resorption of phosphorus from decaying biomass^46^ and loss of phosphorus through leaching^47^. The values of C:P ratio, resorption and leaching vary considerably between different species of lichens and bryophytes. Here we use medium values of these properties^22^, and we assume that today's lichens and bryophytes do not differ much from their Ordovician predecessors in this respect. Finally, the amount of rock weathered by lichens and bryophytes is estimated from their uptake of phosphorus divided by the phosphorus concentration of the rocks. Since we do not know the spatial distribution of rock types at the surface in the Ordovician, we assign a globally uniform phosphorus content to the land surface of 1,432 g per m^3^ rock. This value corresponds to an area-weighted mean rock phosphorus concentration^22^.

Our estimate of weathering would change proportionally for a different value of the mean global rock phosphorus concentration. The value of 1,432 g per m^3^ used here is derived from a recent lithology map^48^. Older estimates of lithology^49^ provide area fractions of rock types not only for today but also for the Ordovician, but probably overweight shallow phosphorus-poor sediments such as sandstones. Using these area fractions would result in 1,232 g phosphorus per m^3^ rock for today and 1,374 g per m^3^ for the Ordovician. Comparing these three values shows that the uncertainty in the mean rock phosphorus concentration is quite small.

### Limits on chemical weathering

In areas where the land surface has a shallow relief and erosion is consequently low, weathering by lichens and bryophytes may become limited by a layer of highly weathered and phosphorus-depleted material blocking the access to unweathered rock substrate. This means that the rate of chemical weathering cannot exceed the rate of exposure of unweathered rock material at the surface, which is equal in the steady state to the erosion rate. We quantify the global distribution of erosion rates as a function of surface elevation above the sea level. We use a method^32^ that relates erosion to the mean relief, and we set the mean relief proportional to the mean surface elevation^50^. Elevation data for the Ordovician are taken from a global palaeoclimate modelling study^34^.

Our method to estimate chemical weathering implies that a corresponding amount of physical weathering takes place to provide sufficient surface area for chemical weathering. We represent this potential limitation of chemical weathering implicitly by calculating the erosion rate. Only material that has been subjected to physical weathering to some extent can be transported by erosion. Hence, the generation rate of fresh rock material by physical weathering, which equals the erosion rate in steady state, sets the upper limit for the rate of chemical weathering in our approach.

Weathering reactions can only occur at mineral surfaces if water that is saturated with the products of weathering is exchanged by precipitation and runoff. Thus, chemical weathering is limited in steady state to the rate of export of dissolved weathering products. We estimate this export of dissolved material by calculating the calcium flux from the soil as a product of runoff and soil calcium concentration, and then divide by the calcium concentration of rocks to obtain the flux of total rock material. To determine soil calcium concentration, we apply an equilibrium approach^51^. The maximum concentration of calcium in soil water is approximated by the equilibrium with respect to calcite. This means that higher concentrations of calcium in the soil solution are prevented by precipitation of calcite. The calcite equilibrium depends on the partial pressure of CO_2_ in the soil and on the mineral composition of the soil or regolith. The latter influences calcium concentration through the relative activity of minerals with and without calcium and the effects of cations other than Ca^2+^ on the charge balance with 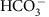. Owing to the uncertain spatial distribution of Ordovician surface rocks, we use a globally uniform mineral composition, based on the area-weighted mean for today's lithology classes^51^. In addition, our estimate of rock calcium concentration is based on this mean mineral composition, assuming a calcium content of 40% for calcite and 14% for calcium-feldspar. Since we lack information about soil CO_2_ concentration in the Ordovician, we assign a globally uniform value of 10,000 p.p.m., which is a realistic value for today's soils at intermediate depths^52^. Note that CO_2_ concentration in the uppermost part of the soil was at least 2,240 p.p.m. in the Ordovician, assuming an atmospheric CO_2_ concentration of 8 PAL.

Runoff is generated in the lichen and bryophyte model when water input by rainfall, dew or snowmelt exceeds the water-storage capacity of the vegetation. We only consider runoff from lichen- and bryophyte-covered areas to contribute to chemical weathering, which leads to underestimation of weathering for regions with sparse vegetation cover. However, we do not expect this to influence strongly our estimates, since abiotic chemical weathering on bare areas is probably several times lower than biotic weathering^14^,^23^. By assuming that only runoff is in contact with mineral surfaces, we potentially further underestimate chemical weathering, since also mineral dust deposited on top of lichens and bryophytes and wetted by rainfall or dewfall may be weathered by the organisms. In the Ordovician, atmospheric dust consisted most probably of unweathered rock particles because of the lack of organisms other than lichens and bryophytes, which were capable of transforming significant amounts of rock phosphorus into organic phosphorus. The dust could therefore represent a source of phosphorus in arid regions, where no runoff is generated and dewfall is the only source of water for lichens and bryophytes.

### Evaluation of erosion- and runoff-limited chemical weathering

We apply our limit-based approach to estimate today's global chemical weathering flux. To be consistent with our estimate for the Ordovician, we use the same globally uniform mean values of soil mineral composition and soil CO_2_ concentration. We determine runoff for each grid cell from the Budyko curve, where we calculate potential evaporation by the equilibrium evaporation approach^53^. Annual mean values of radiation, temperature and precipitation are computed from today's climatic fields^20^. The simulated potential global calcium flux amounts to 10.7e12 mol yr^−1^, which compares well with the 9.3e12 mol yr^−1^ estimated by a more complex model that represents the spatial distribution of lithological classes and includes a process-based scheme for soil CO_2_ (ref. 51). Subsequently, we take the minimum of the runoff-based calcium flux and the flux supported by erosion, where we convert the rock material flux associated with erosion to a potential calcium flux using average mineral properties. This reduces the estimated calcium flux to 7.7e12 mol yr^−1^, which corresponds to a global chemical weathering of 1.8 (km^3^ rock) yr^−1^. This value is around twice the value based on measurements in the world's rivers^54^, converted to rock volume using an average rock density of around 2,500 kg m^−3^. We explain this by two reasons: First, the measurements likely underestimate the rock volume affected by chemical weathering, since some rock types form insoluble clay minerals during weathering, which are not captured by the measurement method (total dissolved solids). Second, kinetic limitation of weathering reactions may be important in some regions, such as mountain areas in the humid tropics, for instance, leading to an overestimation of chemical weathering by the limit-based approach.

To test these potential explanations we apply the GEM-CO2 model^55^ that predicts chemical weathering as a linear function of runoff for different lithology classes. These classes are the same as those used in our approach. On the basis of average mineral properties we estimate a global calcium flux of 5.5e12 mol yr^−1^, which translates into a rock volume of 1.3 (km^3^ rock) yr^−1^. This suggests that clay formation and kinetic limitation contribute roughly equally to the overestimation of chemical weathering by our approach compared with measurements from the world's rivers. Keeping these uncertainties in mind we believe that the erosion- and runoff-based limits on chemical weathering represent a reasonable first approximation to the actual rate of global chemical weathering and should be suitable to constrain our estimate of chemical weathering in the Late Ordovician to realistical values.

To distinguish between biotic and abiotic contributions to the global chemical weathering flux, we quantify Ordovician abiotic weathering using the GEOCARB III model^14^. This means that we scale today's global calcium flux to the Ordovician calcium flux, thereby assuming abiotic conditions in the Ordovician. As a first step, we calculate today's calcium fluxes associated with chemical weathering of silicates as well as carbonates with a spatially explicit version of the GEM-CO2 model, since GEOCARB uses different equations for the two fluxes. We estimate 2.1e12 mol yr^−1^ of carbonate weathering and 3.4e12 mol yr^−1^ of silicate weathering, which matches well with the value of 5.5e12 mol yr^−1^ for the total calcium flux based on average lithology and mineral properties stated above.

The scaling relationship in GEOCARB III consists of several factors, which describe how changes in temperature, runoff and atmospheric CO_2_ between today and the Ordovician influence rates of weathering reactions. We use the standard values from GEOCARB III parameterize these factors and we adopt the derivation of runoff from temperature. We do not, however, use the GEOCARB approach to derive Ordovician surface temperature from atmospheric CO_2_, but instead we use the average land surface temperature estimated by the LMDZ atmospheric circulation model, which is also used to force the non-vascular vegetation model. In this way estimates of both biotic and abiotic weathering are based on the same CO_2_ and temperature forcing. Furthermore, GEOCARB III includes the effects of changes in uplift, land surface area and biotic enhancement of weathering. For uplift, we use the GEOCARB III value for the late Ordovician. The change in land surface area is derived from our forcing data to be consistent with the estimate of biotic weathering. GEOCARB III assumes that the lack of large vascular land plants before the Devonian reduced the global weathering rate to a fraction of 0.25. GEOCARB does not distinguish between non-vascular vegetation and abiotic conditions. Hence, the factor of 0.25 integrates studies that compare weathering below vascular plants to weathering in bare soil as well as studies that compare vascular plants to lichens and bryophytes. For bare soil the factor ranges from 0.25 to 0.1 and, for lichens and bryophytes it amounts to 0.25 based on one study. Since we do not want to underestimate abiotic weathering, we adopt the GEOCARB III standard value of 0.25 for the Ordovician. Finally, we convert the calcium fluxes to rock volume using average rock properties of carbonate and silicate minerals.

We use the GEOCARB approach for estimating abiotic weathering since our erosion- and runoff-limited approach does not contain an explicit description of the influence of biota on weathering. While the original, more complex version of the limit-based approach includes the impact of root respiration on soil CO_2_, the authors point out that many other factors that are important for enhancing the weathering rate are not considered^51^. Among these are release of organic acids, retention of water at the rock surface, break up of rock surface by rhizines and rhizoids and supply of carbon to soil microorganisms that perform weathering. Instead of representing the factors explicitly, the limit-based approach rests on the assumption that the factors reduce the kinetic limitation of the weathering reactions to a large extent. As a consequence, chemical weathering is rather limited by climatic and topographic constraints. The fact that the limit-based approach provides a reasonable approximation of realized chemical weathering suggests that kinetic limitation of weathering is indeed not a decisive factor in large parts of the world, such as flat or dry areas. However, without biota, the kinetic limitation of the weathering reactions probably becomes much more important. For this reason, we think it is more appropriate to use a kinetic approach for abiotic weathering, such as the GEOCARB III model.

### Phosphorus limitation of NPP

In some regions the phosphorus requirement of lichens and bryophytes based on their potential NPP may not be met by the amount of phosphorus available from the substrate, since biotic weathering is limited by low erosion or runoff. In these areas, NPP is phosphorus-limited and we consequently reduce our estimate of NPP by converting the flux of available phosphorus into realized NPP via the C:P ratio of biomass. However, the availability of phosphorus from the substrate may be higher than the amount released by weathering because of additional release of phosphorus by microorganisms that decompose organic matter, or phosphorus remaining in the substrate after leaching. If the residence time of water in the substrate is sufficiently long, this additional phosphorus may be taken up by the organisms, resulting in recycling of phosphorus in addition to the effects of resorption and leaching described above, which almost compensate each other. The availability of phosphorus in the substrate probably depends on spatially varying hydrological conditions: rainfall is the main driver for leaching of phosphorus from the organisms, while runoff is necessary to export phosphorus from the substrate. Therefore, phosphorus availability should be lower in humid regions. However, water is necessary for the decomposition of organic matter, which releases phosphorus, leading to lower phosphorus availability in arid regions. In today's ecosystems, these effects are probably strongly modulated by the effect of roots of vascular plants on soil water-residence time. For simplicity, we assume here a global uniform value of the recycling of phosphorus in the substrate.

The recycling ratio for phosphorus can be estimated by dividing the phosphorus uptake associated with NPP by the phosphorus release from weathering. Since we want to estimate the recycling ratio for the Late Ordovician, we consider those of today's ecosystems, which are dominated by lichens and bryophytes, such as deserts. Typical values for NPP of lichens and bryophytes in deserts are ∼5 (g C) m^−2^ yr^−1^, although they show large spatial variation^20^. Weathering by lichens and bryophytes on rock surfaces^9^ ranges from 0.0004 to 0.01 mm yr^−1^. Assuming a typical value of 0.002 mm yr^−1^, the mean values for C:P ratio and rock phosphorus concentration, we calculate a recycling ratio of ∼5:1.

We run the lichen and bryophyte model with and without nutrient limitation for today's climate. We assume a recycling ratio of 5:1 for bare soil, but for regions that are dominated by vascular vegetation we use a recycling ratio of 50:1 (ref. 30). This high recycling ratio lowers the phosphorus requirement of lichens and bryophytes below the amount provided by erosion- or runoff-limited chemical weathering; the reduction of potential NPP thus mostly occurs in desert regions. At the global scale, NPP is reduced by 16% from 3.1 to 2.6 (Gt C) yr^−1^. In dry areas, the reduction of NPP reduces the average value for the desert biome from 13.7 to 6.2 (g C) m^−2^ yr^−1^, which improves the model fit to the typical observed value of 5 (g C) m^−2^ yr^−1^ estimated in a previous study^20^. Note that simulated chemical weathering rates of today's lichens and bryophytes in desert regions (Fig. 5a) compare well with observed rates on bare rocks^9^.

### Sensitivity analyses

We perform a sensitivity analysis to assess the relevance of several uncertainties, which are associated with our modelling approach. To account for the considerable uncertainty regarding the value of Ordovician atmospheric CO_2_ content^3^, we force the lichen and bryophyte model with additional LMDZ climatic fields corresponding to levels of atmospheric CO_2_ of 3, 4, 6, 10, 12, 16 and 24 PAL (see Fig. 2a).

We further analyse the sensitivity of simulated NPP to ambient CO_2_ concentrations by running the model for individual artificial species for a range of ambient CO_2_ concentrations assuming constant light, temperature and water content. We test one species well adapted to today's climate and another one well adapted to Ordovician climate with an atmospheric CO_2_ concentration of 24 PAL (Fig. 4a). To find well-adapted species, we select two regions with similar climatic conditions, one for the Ordovician at 24 PAL and one for today's climate, and use the parameters of the dominant species in these regions. NPP is calculated in the model as the difference of GPP and respiration. GPP is simulated by the well-established Farquhar scheme, which quantifies photosynthesis as a function of light, temperature and CO_2_ concentration within the chloroplasts. The latter depends on diffusion of CO_2_ from the atmosphere into the thallus, which is controlled in the model by the limiting effect of water content on CO_2_ diffusivity. The Farquhar scheme depends on several physiological parameters, such as the carboxylation rate of Rubisco, for instance, which vary considerably between the different artificial species simulated by the model. In addition, the relation between thallus CO_2_ diffusivity and water content is species-specific. At constant temperature and water content, respiration is constant in the model and it does not depend on CO_2_. Therefore, the response of NPP to CO_2_ is driven by GPP. In addition to atmospheric CO_2_, weak photorespiration because of low atmospheric O_2_ may lead to high GPP in Ordovician climate conditions.

The strong CO_2_ sensitivity of the lichen and bryophyte model begs the question: is there experimental evidence for a large increase in NPP because of CO_2_ fertilization? We reviewed studies where lichens and bryophytes were treated with elevated ambient CO_2_ levels and their growth was measured. While some studies did not find any significant effects^56^,^57^,^58^, others found partly strong increases in NPP at elevated CO_2_ (refs 59, 60, 61, 62, 63, 64, 65). Figure 4b shows a summary of those studies.

In some cases where NPP did not increase at elevated levels of ambient CO_2_ or decreased again after a longer period of fertilization, phosphorus limitation was suggested as an explanation^56^,^57^. These studies were, however, conducted with moss growing in peatlands or similar environments, where the access to rock substrates is limited. Lichens and bryophytes growing on bare rock surfaces are capable of taking up elements from the rocks^1^. In this case, enough phosphorus could be available to alleviate nutrient limitation of NPP.

**Figure 7:**
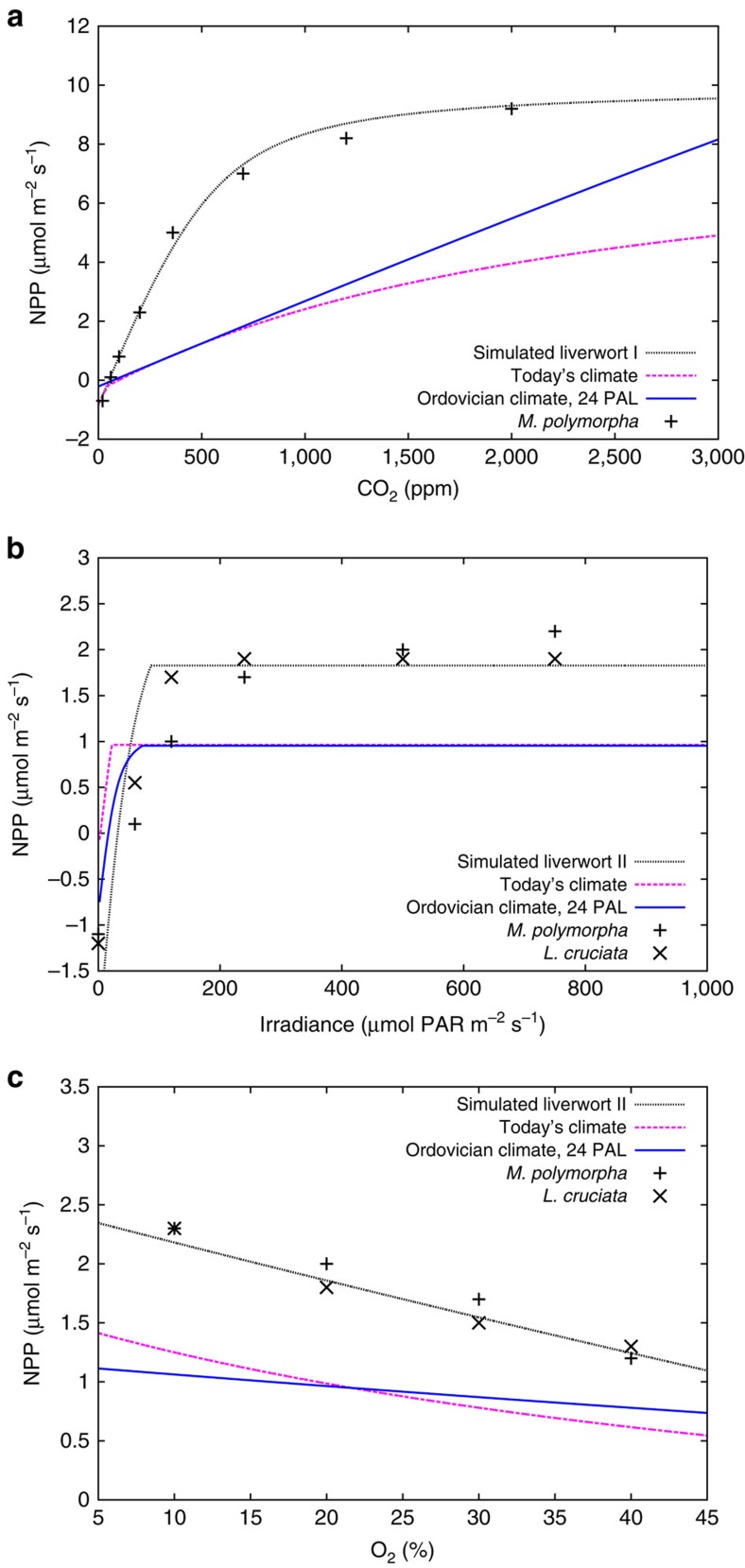
Response of NPP to environmental conditions. NPP calculated by the model for saturated water content and a temperature of 20 °C at varying levels of CO_2_, light and O_2_ compared with observational data from a study on liverworts^66^. The black dotted line correspond to an artificial species parameterized to reproduce the observational data. Two versions of this species, I and II, are used to take into account that the liverworts on which the data points are based differ in photosynthetic capacity. The blue solid line corresponds to an artificial species adapted to Ordovician climate and an atmospheric CO_2_ concentration of 24 PAL, the dashed magenta line corresponds to an artificial species adapted to today's climate and CO_2_ concentration. (**a**) CO_2_ response of our model for an irradiance of 250 μmol m^−2^ s^−1^ of photosynthetically active radiation and an ambient O_2_ concentration of 21%. (**b**) Light response of the model for ambient concentrations of 400 p.p.m. of CO_2_ and 21% of O_2_. (**c**) O_2_ response at an irradiance of 250 μmol m^−2^ s^−1^ and a CO_2_ concentration of 400 p.p.m.

To further test model performance, we compare simulated sensitivity of NPP to varying levels of ambient CO_2_, light and O_2_ to observations from a study on liverworts^66^. In Fig. 7 we show that the model is able to reproduce the CO_2_, light and O_2_ responses of the liverworts. The observational data for the light and O_2_ response curves were obtained from two species, *Marchantia polymorpha* and *Lunularia cruciata*, whereas the CO_2_ response includes only *M. polymorpha*, Furthermore, the observed NPP values in Fig. 7a are based on liverworts grown under different conditions than the liverworts of which NPP measurements are shown in Fig. 7b,c. While laboratory-grown *M. polymorpha* has an NPP of ∼5 μmol m^−2^ s^−1^ at 400 p.p.m. and 250 μmol m^−2^ s^−1^ of photosynthetically active radiation, *M. polymorpha* grown outdoors has an NPP of ∼2 μmol m^−2^ s^−1^ for the same CO_2_ and light forcing (compare Fig. 7a,b). Hence, we use two different parameterizations for our model, which mainly differ in photosynthetic capacity, to reproduce the observational data in Fig. 7. Moreover, we include in Fig. 7 the CO_2_, light and O_2_ response of the two artificial species from Fig. 4a for comparison. The artificial species have a lower NPP than the liverworts at the CO_2_ level of 400 p.p.m., but their relative increase in NPP from 400 p.p.m. of CO_2_ to 1,000 p.p.m. is larger than the liverworts' (Fig. 7a). This can be explained largely by the lower photosynthetic capacity of the two artificial species from Fig. 4a compared with the simulated liverworts; their NPP saturates only at higher levels of ambient CO_2_. In addition, other parameters have an effect, such as thallus diffusivity for CO_2_ and parameters, which control the temperature response of photosynthesis. The latter plays a role since the regions from which the two artificial species have been selected exhibit different temperature and light conditions than the environment in which the liverworts were grown.

The existence of a continental-sized ice sheet south of 30° S in the Hirnantian Stage is debated^24^, which would strongly reduce the available area for growth of lichens and bryophytes. Hence, we run a second baseline simulation with a prescribed glacier mask and updated climatic fields accounting for the presence of the ice sheet. The outcome of this sensitivity analysis is shown in (Fig. 2b).

We test the sensitivity of our estimates to the chosen globally uniform mean values of soil CO_2_ concentration and mineral composition. For a soil CO_2_ concentration of 3,000 p.p.m., weathering decreases by 18% to 2.3 (km^3^ rock) yr^−1^ because of reduced runoff-limited chemical weathering and NPP decreases equally by 17% to 11.9 (Gt C) yr^−1^ as a result of phosphorus limitation. Conversely, at 30,000 p.p.m. of soil CO_2_, weathering increases by 7% to 3.0 (km^3^ rock) yr^−1^ and NPP increases equally by 8% to 15.5 (Gt C) yr^−1^. Mineral composition controls the equilibrium calcium concentration in the soil as well as the calcium content of the minerals. Consequently, it modulates the relation between runoff and the chemical weathering flux. We assign extreme values for mineral composition based on a map of today's lithology^48^, which contains six lithological classes: sandstone, limestone, shale, granite, rhyolite and basalt. Chemical weathering at a given runoff and soil CO_2_ concentration is maximal for granite; therefore, weathering and NPP increase by ∼15% to 3.2 (km^3^ rock) yr^−1^ and 16.6 (Gt C) yr^−1^, respectively. For shale, chemical weathering is minimal, which leads to a reduction of weathering and NPP by ∼40% to 1.6 (km^3^ rock) yr^−1^ and 8.3 (Gt C) yr^−1^, respectively.

In addition to environmental factors such as atmospheric CO_2_, ice sheets and substrate properties, we test the sensitivity of the model with regard to the following model parameters, which have been shown to considerably influence simulated NPP^20^ and which might have changed from the Ordovician to today: disturbance interval, diffusivity of the water-saturated lichen or bryophyte thallus for CO_2_, ratio of Rubisco content to maintenance respiration at a given temperature and efficiency of the carbon concentration mechanism (CCM; Table 2).

We know very little about the disturbance regime in the Ordovician, hence we chose strongly different disturbance intervals 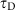, from 10 years to 250 years. The effect on NPP and, consequently, weathering, however, is not large. Only cover and biomass are significantly reduced by frequent disturbances. The ratio of Rubisco content to maintenance respiration at a given temperature Φ_RR_ controls how much organisms have to pay in form of maintenance respiration to achieve a certain photosynthetic capacity, which is determined by the Rubisco content. High photosynthetic capacity represents a competitive advantage in high-light environments because of high potential growth, but under low-light conditions it may have negative effects because of the associated high respiration. For a doubling of Φ_RR_, weathering and NPP increase by ∼20% and 30%, respectively, whereas halving Φ_RR_ leads to a decrease of 17 and 26%. Φ_RR_ has the strongest impact on the model. The efficiency of the CCM *η*_CCM_ determines by which factor the organisms can increase their internal CO_2_ concentration above the ambient level through channelling off electrons from the light-dependent reactions of photosynthesis. While the baseline scenario uses a value of 8, which leads to realistic patterns of NPP for today's lichens and bryophytes, *η*_CCM_ is varied from a low factor of 2 to the theoretical factor of 45 based on a study in cyanobacteria^67^. It has a weaker impact on NPP than Φ_RR_. Φ_RR_ and *η*_CCM_ have the same value for each species since they represent universal physiological constraints^20^. The diffusivity of the water-saturated lichen or bryophyte thallus for CO_2_, 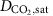, however, does not have a uniform value but is sampled for each species from the observed range of values. 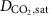 does not represent a physiological constraint by itself, but it is part of a tradeoff between the influx of CO_2_ into the thallus and resistance to water loss. A dense thallus prevents rapid water loss by evaporation, but it also hampers high influx of CO_2_. Thus, we extend the range once by doubling the maximum 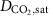 and once by halving the minimum 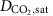. The effect on NPP is similar to that of *η*_CCM_. To summarize, the estimates seem to be robust over a wide range of physiological parameter values.

At low numbers of artificial species, the lichen and bryophyte model may underestimate NPP since the random sample may be too small to contain combinations of physiological parameter values leading to high NPP. We run simulations with initial numbers of 10, 30, 100, 300, 1,000 and 3,000 artificial species to determine at which number of species physiological variation is represented to a sufficient degree, so that NPP and weathering remain constant thereafter. The number of 300 initial species chosen for the baseline scenario seems to be sufficient to represent the physiological variation of the organisms (Fig. 3).

### Code availability

The non-vascular vegetation model used in this study is integrated into an interface for parallel computing, which was developed at the Max Planck Institute for Biogeochemistry, Jena, Germany. In addition to the model, a post-processing script is necessary to derive the results presented in this study from the model output. The model, the interface and the script are freely available as long as the copyright holders and a disclaimer are distributed along with the code in source or binary form. The code is available from the corresponding author upon request.

### Data availability

The findings in this study are based on climatic fields simulated by the atmospheric general circulation model LMDZ^41^. Requests for the climate model output can be sent to A.P (alexandre.pohl@lsce.ipsl.fr) or Y.D. (yannick.donnadieu@lsce.ipsl.fr).

## Additional information

**How to cite this article:** Porada, P. *et al*. High potential for weathering and climate effects of non-vascular vegetation in the Late Ordovician. *Nat. Commun.* 7:12113 doi: 10.1038/ncomms12113 (2016).

## Supplementary Material

Supplementary Information Supplementary Figure 1, Supplementary Note 1 and Supplementary References.

## Figures and Tables

**Table 1:** Global effects of lichens and bryophytes.

**Simulation**	**NPP (Gt C) yr**^**−1**^	**GPP (Gt C) yr**^**−1**^	**Cover fraction**	**Biomass Gt C**	**Weathering (km**^**3**^ **rock) yr**^**−1**^
Ordovician	14.4	30.1	0.44	133	2.8
Today					
Lichens and bryophytes	2.6	5.4	0.14	51	0.044
Total vegetation	60 (ref. 25)	123 (ref. 68)	0.74 (ref. 69)	442 (ref. 70)	0.85 (ref. 54)

GPP, gross primary productivity; Gt C, gigatons of carbon; NPP, net primary productivity.

Annual global values of NPP, GPP, surface coverage, biomass and weathering for the Ordovician baseline simulation (see Methods section) and for today's climate. Note that we converted the weathering estimate^54^ by using an average density of surface rocks^22^.

**Table 2:** Sensitivity to model parameters.

**Scenario**	**NPP ((Gt C) yr**^**−1**^**)**	**GPP ((Gt C) yr**^**−1**^**)**	**Cover fraction**	**Biomass (Gt C)**	**Weathering ((km**^**3**^ **rock) yr**^**−1**^**)**
Φ_*RR*_ low	10	23	0.4	102	2.3
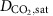 low	12	25	0.4	124	2.5
*η*_CCM_ Low	13	27	0.4	126	2.6
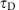 250 yr	14	31	0.5	170	2.8
Baseline	14	30	0.4	133	2.8
*η*_CCM_ high	15	31	0.4	135	2.9
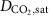 High	15	31	0.4	134	2.9
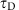 10 yr	15	27	0.2	40	3.0
Φ_RR_ High	19	38	0.4	153	3.4

GPP, gross primary productivity; Gt C, gigatons of carbon; NPP, net primary productivity.

Global annual values of NPP, GPP, surface coverage, biomass and weathering for the Ordovician baseline simulation (see Methods section) and for upper and lower bounds of four key model parameters. See text for a description of the parameters and the associated upper and lower bounds.
