# Supplementary material for: High potential for weathering and climate effects of non-vascular vegetation in the Late Ordovician
Source: Nat Commun. 2016 Jul 7;7:12113. doi: 10.1038/ncomms12113 (PMC4941054; doi:10.1038/ncomms12113)
Supplement: Supplementary Information — Supplementary Figure 1, Supplementary Note 1 and Supplementary References. [file ncomms12113-s1.pdf]

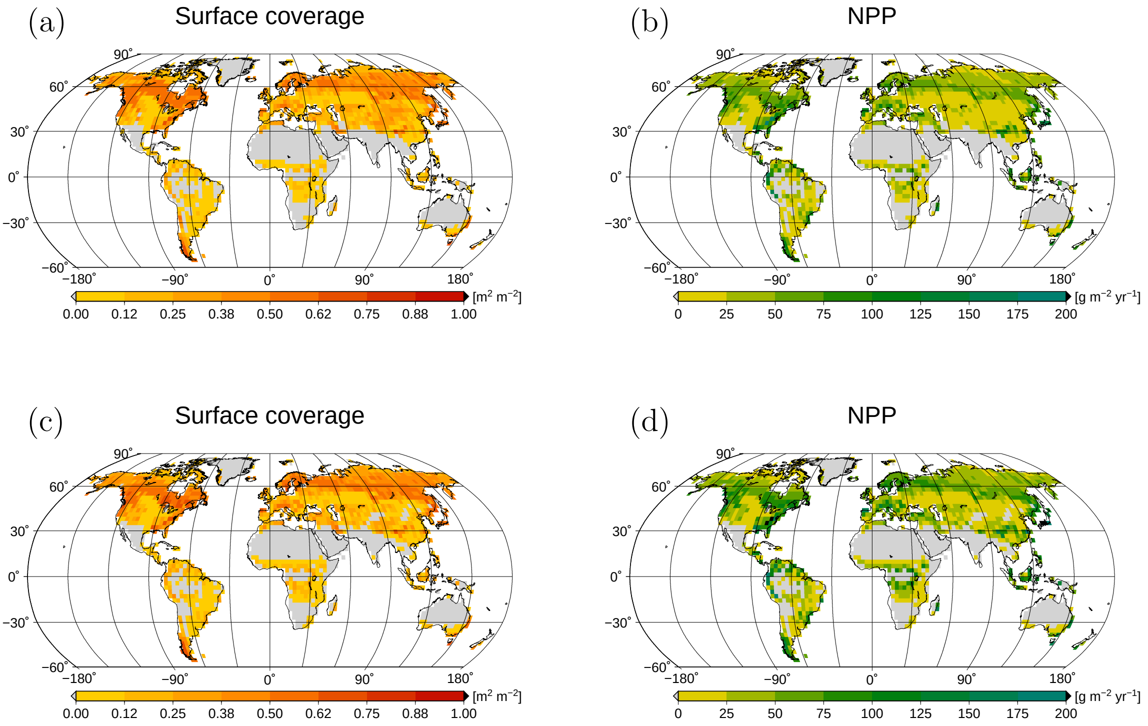

Supplementary Fig. 1. Effect of generated climatic fields on NPP and surface coverage Global patterns of NPP and surface coverage estimated by the lichen and bryophyte model for today's climate, in the presence of vascular plants. a) and b) correspond to a simulation forced with the original hourly climatic fields[1]. c) and d) correspond to a simulation forced with hourly climatic fields generated by the weather generator. The maps show average values over the last 50 years of a 600 year simulation with 300 initial species.

Supplementary Note 1. Weather generator

The weather generator combines several established methods of interpolation of climate variables: In a first step, rainfall and snowfall are disaggregated from monthly to daily values using a Markov process for the distribution of wet days and an exponential distribution of precipitation amount[2]. The other daily climate variables are set to the respective monthly value for simplicity.

To disaggregate daily into hourly rainfall we proceed as follows[3]: The number of rainfall events on any wet day is sampled from a poisson distribution, their duration and the amount of rainfall per event are sampled from exponential distributions. For the starting time of each event, we approximate the original beta cumulative distribution function[3] by a linear one. For rainfall events that last longer than one hour, an exponential decrease of rainfall intensity during the event is assumed while the intensity of the first hour follows a log-normal distribution[4]. For the disaggregation of daily into

hourly snowfall, the same method is applied. The distribution of shortwave radiation on a given day is described as a sine wave between sunrise and sundown, with a maximum at noon, while daylength is determined by the calendar day[5]. Downwelling longwave radiation is assumed to be evenly distributed over the whole day for simplicity. The diurnal course of air temperature is computed via the daily minimum and maximum air temperatures, provided as monthly values by LMDZ. By assuming that minimum air temperature occurs directly before sunrise and maximum air temperature two hours after solar noon, a sine wave is fitted between the minimum and maximum temperature values to obtain hourly air temperature[6]. To estimate hourly values of relative humidity, it is assumed that the respective minimum value occurs at sunrise due to dewfall throughout the night[7]. Thus, minimum relative humidity is computed as a function of daily minimum air temperature and the saturation water vapour pressure[8]. Through evaporation, relative humidity then increases during the day until sundown, when it starts decreasing again. We use a linear function to connect the minima and maxima of subsequent days, where the maximum daily relative humidity is fitted so that the average humidity over the day matches the daily value from LMDZ. Hourly values of wind speed were derived from daily values using a cosine function according to[9].

To test the performance of the weather generator, the lichen and bryophyte model is run with a multi-year hourly climatic field set[1]. Then, the hourly fields are averaged to one year of monthly values and these values are interpolated back to hourly fields via the weather generator. A second simulation is then run with these synthetic climate fields and the results are compared to those of the first simulation. The patterns of NPP and surface coverage look similar (Supplementary Fig. 1) and their global values do not differ much, amounting to a coverage of 17 % and an NPP of 3.9 Gt yr^{-1} for the synthetic fields and 16 % and 3.1 Gt yr^{-1} for the original fields, respectively. Thus, we conclude that the hourly climatic fields produced by the weather generator are sufficiently realistic for the purpose of our study.

References

1. Porada, P., Weber, B., Elbert, W., Pöschl, U. & Kleidon, A. Estimating global carbon uptake by lichens and bryophytes with a process-based model. *Biogeosciences* **10,** 6989–7033 (2013).
2. Richardson, C. Stochastic simulation of daily precipitation, temperature and solar radiation. *Water Resources Research* **17,** 182–190 (1981).
3. Connolly, R., Schirmer, J. & Dunn, P. A daily rainfall disaggregation model. *Agricultural and Forest Meteorology* **92,** 105–117 (1998).
4. Maréchal, D. & Holman, I. A robust and parsimonious regional disaggregation method for deriving hourly rainfall intensities for the UK. *Hydrology and Earth System Sciences Discussions* **2,** 1047–1065 (2006).
5. *Principles of Environmental Physics (Fourth Edition)* (eds Monteith, J. & Unsworth, M.) (Academic Press, Boston, 2013).

6. *An Introduction to Environmental Biophysics* (eds Campbell, G. & Norman, J.) (Springer, New York, 1998).

7. Kimball, B. & Bellamy, L. Generation of diurnal solar radiation, temperature, and humidity patterns. *Energy in Agriculture* **5,** 185–197 (1986).

8. Monteith, J. Evaporation and surface temperature. *Quart. J. R. Met. Soc.* **107,** 1–27 (1981).

9. Guo, Z. A simple method to downscale daily wind statistics to hourly wind data. *arXiv preprint arXiv:1305.3367* (2013).
